# Editorial: Reviews and Novel Clinical Perspectives on Semaglutide: A GLP-1 Receptor Agonist With Both Injectable and Oral Formulations

**DOI:** 10.3389/fendo.2021.760153

**Published:** 2021-09-23

**Authors:** Juris J. Meier, Baptist Gallwitz, Francesco Giorgino

**Affiliations:** ^1^ Department of Internal Medicine, Gastroenterology and Diabetology, Augusta Clinic, Bochum, Germany; ^2^ Department of Medicine IV - Diabetes, Endocrinology, Nephrology, Tübingen University Hospital, Tübingen, Germany; ^3^ Department of Emergency and Organ Transplantation, University of Bari Aldo Moro, Bari, Italy

**Keywords:** glucagon-like peptide-1 receptor agonist, oral, semaglutide, type 2 diabetes, subcutaneous

The potential of glucagon-like peptide-1 (GLP-1) as a therapeutic target in type 2 diabetes (T2D) was first realized with the discovery that GLP-1 plays a key role in augmenting insulin secretion in response to nutrient intake (1). Subsequently, GLP-1 receptor agonists (GLP−1RAs) have been shown to increase insulin and decrease glucagon secretion in a glucose-dependent manner, resulting in reduced blood glucose levels, but with a low risk of hypoglycemia. GLP-1RAs also improve multiple pathophysiological defects in T2D beyond glycemic control, including reduction of body weight. Several cardiovascular (CV) outcomes studies have also shown that some GLP-1 RAs, namely liraglutide, semaglutide, and dulaglutide, can effectively prevent CV events, such as acute myocardial infarction or stroke, and associated mortality (2).

Although GLP-1RAs act *via* the same overall mechanism, they vary structurally and in their pharmacokinetic and clinical effects. Early GLP-1RAs needed to be administered subcutaneously (s.c.) once or twice daily. To reduce the injection burden and improve convenience, molecules and formulations were modified to create GLP-1RAs that require less frequent administration. Semaglutide is one such long-acting GLP-1RA – it shares 94% sequence homology with GLP-1, but three structural modifications extend its half-life to ~1 week, which permits once-weekly s.c. administration (3, 4).

An oral GLP-1RA formulation may be preferred by some patients; however, oral delivery of peptides is difficult due to extensive degradation by proteolytic enzymes in the gastrointestinal tract and poor absorption across the gastrointestinal epithelium. By co−formulating semaglutide with the absorption enhancer, sodium N-(8-[2-hydroxybenzoyl] amino) caprylate (SNAC), a novel oral formulation of semaglutide has been developed. SNAC protects semaglutide against enzymatic degradation *via* a local pH buffering effect and promotes absorption of semaglutide across the gastric epithelium in a concentration-dependent manner by effects on transcellular pathways, which are transient and fully reversible (5). The long half-life of semaglutide helps maintain exposure in the event of any variation in day-to-day absorption of the oral formulation.

This Research Topic discusses the efficacy, general safety, CV effects, and additional clinical perspectives related to semaglutide, in both its s.c. and oral formulations. The review article by Meier describes data on glucose-lowering and body-weight reductions from the SUSTAIN and PIONEER global clinical trial programs that established the efficacy of s.c. and oral semaglutide, respectively, in a range of clinical settings. Factors that may influence the choice of formulation in individual patients are also discussed. In the SUSTAIN and PIONEER programs, s.c. and oral semaglutide were well tolerated, with a long-term safety profile consistent with other GLP−1RAs. The most common adverse events and selected adverse events of interest are described by Smits and Van Raalte, alongside a discussion of mechanistic studies.

The CV safety of s.c. and oral semaglutide have been confirmed in specific CV outcomes trials. The review article by Nauck and Quast summarizes data on CV safety and discusses mechanisms responsible for the CV benefits seen with some GLP-1RAs, including semaglutide, with particular focus on effects related to reversing atherosclerosis, inflammation, and endothelial dysfunction.

Although early use is advocated by international diabetes guidelines, GLP-1RAs are often underutilized. The article by Gallwitz and Giorgino reviews the current place of GLP−1RAs in therapy, and recommendations by medical and scientific societies such as the American Diabetes Association and the European Association for the Study of Diabetes. In addition, the article highlights some clinical considerations related to the use of semaglutide, such as dosing considerations, use in special populations, and ongoing large-scale studies that will add to the evidence base of s.c. and oral semaglutide in T2D, and potentially contribute to new indications.

## Author Contributions

The authors were involved with drafting and/or critically reviewing all drafts during the development of the article, and provided final approval for submission. All authors contributed to the article and approved the submitted version.

## Funding

This article was supported by Novo Nordisk, who was provided with the opportunity to perform a medical accuracy review.

## Conflict of Interest

JM has received lecture honoraria and consulting fees from AstraZeneca, Berlin-Chemie, Boehringer Ingelheim, Eli Lilly, Merck Sharp & Dohme, Novartis, Novo Nordisk, and Sanofi; has received reimbursement of congress participation fees and travel expenses from Merck Sharp & Dohme, Novo Nordisk, and Sanofi; and has initiated projects supported by Boehringer Ingelheim, Merck Sharp & Dohme, Novo Nordisk, and Sanofi. FG has received research support from Eli Lilly, Lifescan, and Takeda; and has provided advisory services to AstraZeneca, Boehringer Ingelheim, Eli Lilly, Lifescan, Merck Sharp & Dohme, Novo Nordisk, Roche Diabetes Care, and Sanofi. BG has received lecture honoraria and provided advisory services to AstraZeneca, Boehringer Ingelheim, Eli Lilly, Merck Sharp & Dohme, and Novo Nordisk; and has received lecture honoraria from Bristol Myers Squibb.

The author declares that this article received funding from Novo Nordisk. The funder had the following involvement in the article: medical writing support.

## Publisher’s Note

All claims expressed in this article are solely those of the authors and do not necessarily represent those of their affiliated organizations, or those of the publisher, the editors and the reviewers. Any product that may be evaluated in this article, or claim that may be made by its manufacturer, is not guaranteed or endorsed by the publisher.
